# Facile Synthesis of NiCo_2_O_4_ Nanowire Arrays/Few-Layered Ti_3_C_2_-MXene Composite as Binder-Free Electrode for High-Performance Supercapacitors

**DOI:** 10.3390/molecules27196452

**Published:** 2022-09-30

**Authors:** Yanhua Li, Shuhuan Wang, Guolong Ni, Qun Li

**Affiliations:** 1 School of Metallurgy and Energy, North China University of Science and Technology, Tangshan 063210, China; 2 Key Laboratory of Special Metallurgy and Material Manufacture, Tangshan 063210, China

**Keywords:** Ti_3_C_2_ nanosheets, NiCo_2_O_4_ nanowires, composites, asymmetric supercapacitor, energy density

## Abstract

Herein, a 3D hierarchical structure is constructed by growing NiCo_2_O_4_ nanowires on few-layer Ti_3_C_2_ nanosheets using Ni foam (NF) as substrate via simple vacuum filtration and solvothermal treatment. Ti_3_C_2_ nanosheets are directly anchored on NF surface without binders or surfactants, and NiCo_2_O_4_ nanowires composed of about 15 nm nanoparticles uniformly grow on Ti_3_C_2_/NF skeleton, which can provide abundant active sites and ion diffusion pathways for enhancing electrochemical performance. Benefiting from the unique structure feature and the synergistic effects of active materials, NiCo_2_O_4_/Ti_3_C_2_ exhibits a high specific capacitance of 2468 F g^−1^ at a current density of 0.5 A g^−1^ and a good rate performance. Based on this, an asymmetric supercapacitor (ASC) based on NiCo_2_O_4_/Ti_3_C_2_ as positive electrode and activated carbon (AC)/NF as negative electrode is assembled. The ASC achieves a high specific capacitance of 253 F g^−1^ at 1 A g^−1^ along with 91.5% retention over 10,000 cycles at 15 A g^−1^. Furthermore, the ACS presents an outstanding energy density of 90 Wh kg^−1^ at the power density of 2880 W kg^−1^. This work provides promising guidance for the fabrication of binder-free, free-standing and hierarchical composites for energy storage application.

## 1. Introduction

With climate change and fossil energy depletion of the world, the development of high-performance energy conversion and storage devices is urgently demanded [1,2]. Supercapacitors (SCs) are considered as a new type of promising candidate devices and have been extensively developed due to their high power density, fast charging/discharging rate and long cycling life compared with batteries [3,4]. However, the relatively lower energy density of SCs restricts their development and actual application. It is well known that the energy density (*E*) is related to working potential range (Δ*V*) and specific capacitance (*C*) according to the formula of *E* = *C* (Δ*V*)^2^/2 and the electrochemical behavior of electrode materials plays an important role in performance of SCs [5,6]. Thus, exploring suitable electrode material is promising for improving the energy density and performance of SCs.

MXene (Ti_3_C_2_), a recently novel emerging 2D material, has been widely studied in SCs by virtue of its unique properties, such as high conductivity, unique layered structure, excellent theoretical capacitance energy (1500 F cm^−3^), good mechanical stability and environmentally friendly characteristics [7,8,9]. Moreover, recent researches have proven that MXene shows much promise over other supercapacitor electrode materials and is one of the prospective candidates [10]. Particularly, few-layer Ti_3_C_2_ nanosheets can facilitate the full exposure of O-functionalized surface, which will expedite the accessibility of ions and promote the sufficiently utilization of their functional surfaces [11,12]. However, the MXene materials usually exist in powdery form and must be mixed with conductive carbon and a polymeric binder, followed by painting and pressing on a current collector, when they are used to assemble SCs. The use of binder will significantly deteriorate the electrical conductivity of the active materials and increase the mass of electrode, which is not beneficial to the electrochemical performance [13,14]. Thus, developing free-standing and binder-free electrodes is an effective strategy to avoid the limitations. Recently, Guo et al. [15] reported a composite electrode comprised of 2D delaminated Ti_3_C_2_ sheets (d-Ti_3_C_2_) and 3D Ni foam (NF) by electrostatic self-assembly, and it exhibits a specific capacitance up to 654 F g^−1^ at 1 A g^−1^ and a good cycling stability. Similarly, Hu et al. [16] fabricated the CSC@Ti_3_C_2_Tx electrode via dropping the Ti_3_C_2_T_x_ colloidal suspension with a pipette on the carbonized silk cloth (CSC) strip that was placed on a hot plate kept at 50 °C, and it possesses an area capacitance of 362 mF cm^−2^ with an excellent cyclability and flexibility. However, the above fabrication processes are complicate and time-consuming, such as they need surfactant modification and heating treatment, respectively. Inspired by the advantages of MXene and 3D scaffold, the MXene/NF was synthesized by simple vacuum filtration treatment in this work. Furthermore, the capacitance performance of MXene/NF is still needed to be enhanced for further practical application. At present, the general strategy to solve this problem is to develop suitable synthetic approach to modify MXene with pseudocapacitance electrode materials (such as conducting polymer, layered double hydroxide (LDH) and transition-metal oxides/sulfides) [17], which generally possess higher energy density and specific capacitance than pure carbon materials [18,19].

As the representative of metal oxide, NiCo_2_O_4_ is one of the most successful pseudocapacitive materials used in supercapacitors due to its low cost, various morphologies and structures, environmental benignity and simple preparation [20]. Importantly, NiCo_2_O_4_ can also exhibit extremely higher theoretical specific capacitance in alkaline electrolyte owing to the redox reactions originating from two groups of active centers on Ni ions and Co ions [21,22]. Moreover, the nanostructured NiCo_2_O_4_ is conducive to providing large specific surface area, higher storage site utilization and short ion diffusion path. However, the low electronic conductivity and serious agglomeration hinder its electrochemical performance [23]. A popular approach to avoid the limitation is to develop effective synthetic strategies and design composite materials by combining them with other high conductivity materials. Recently, some researches about the combination of MXene with binary metal hydroxides/sulfides to form composites to improve the capacitance of SCs, in which their integration indeed improves the capacitive performance compared with individual metal hydroxides/sulfides and MXene. Thus, it can be inferred that MXene as a favorable conductive substrate to integrate with NiCo_2_O_4_ for constructing composites is a potential route to enhancing the capacitance of SCs. Furthermore, morphology factor plays an important role in SCs, and the 3D structure not only contributes to enhance ion diffusion kinetics, but avoid the existence of polymer binder, which is conducive to improve performance of electrodes [21,22]. Based on this, if nanostructured NiCo_2_O_4_ is directly combined with 3D MXene materials, the resulting composite materials not only improve the conductivity and more effectively prevent the agglomeration of nanostructured NiCo_2_O_4_, but also will exhibit considerable electrochemical performance by combing the above two advantages, which has profound meaning. As far as is known, the research about NiCo_2_O_4_/Ti_3_C_2_ composites is scant, and they usually exist in powdery form. For instance, Wang at al. reported that the NiCo_2_O_4_/Ti_3_C_2_ powder without self-standing substrate shows a low specific capacitance of 714 F g^−1^ and insufficient capacity retention of 87.8% upon 5000 cycles [24]. However, coupling nanostructured NiCo_2_O_4_ with MXene to form a 3D structure as an electrode for high performance supercapacitors has not been reported, to our knowledge.

Herein, a free-standing and binder-free electrode based on NiCo_2_O_4_/Ti_3_C_2_ nanocomposite using Ni foam as substrate was constructed by a two-step approach including vacuum filtration and solvothermal treatment. The fabrication process is fast and easily operated. The ions and electron transfer will be accelerated and the interfacial resistance in NiCo_2_O_4_/Ti_3_C_2_ will be reduced due to the direct contact of active materials. Benefiting from the unique structure, the specific capacitance of NiCo_2_O_4_/Ti_3_C_2_ can reach up to 2468 F g^−1^ at the current density of 0.5 A g^−1^. Moreover, the electrode exhibits a moderate rate performance. In addition, the assembled NiCo_2_O_4_/Ti_3_C_2_//AC asymmetrical supercapacitor (ASC) exhibits a specific capacitance of 253 F g^−1^ at 1 A g^−1^ and 135 F g^−1^ at 10 A g^−1^ along with 91.5% retention for 10,000 cycles. Surprisingly, the ACS achieves an outstanding energy density of 90 Wh kg^−1^ at a power density of 2880 W kg^−1^, which indicates its huge potential in the field of SCs.

## 2. Experimental Section

### 2.1. Materials

Ti_3_AlC_2_ was sourced from Laizhou Kai Kai Ceramic Materials Co., Ltd., Laizhou, China. Ni foam (NF) was obtained from Guangdong Canrd New Energy Technology Co., Ltd., Dongguan, China. The other reagents, including deionized water, nickel nitrate hexahydrate, cobalt nitrate hexahydrate, urea, acetone, hydrochloride, ethanol, potassium hydrate, N-dimethylmethacrylamide, were bought from Shanghai Macklin Biochemical Co., Ltd., Shanghai, China. Activated carbon (AC) were achieved from Fuzhou Yihuan Carbon Co., Ltd., Fuzhou, China. Polyvinylidene fluoride (PVDF) binder and acetylene black were gained from Beijing Chemical Co., Ltd., Beijing, China. All chemicals used were of analytical grade. Deionized water was used throughout the work.

### 2.2. Synthesis of NiCo_2_O_4_/Ti_3_C_2_ Heterostructure

**Figure 1:**
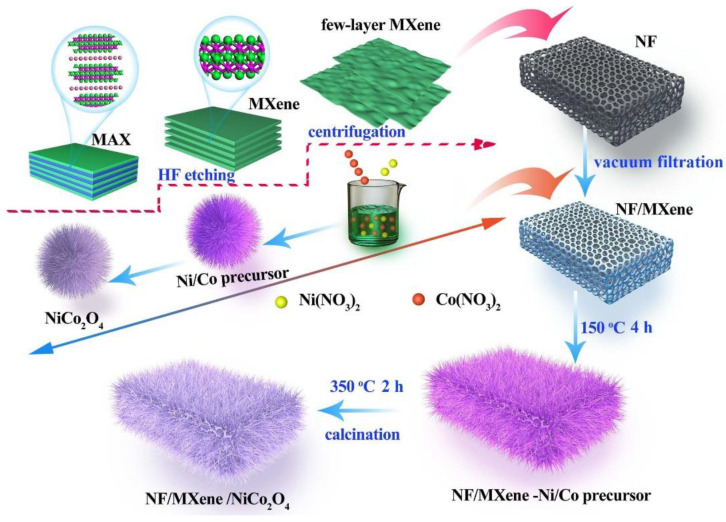
Schematic illustration of the synthesis process of NiCo_2_O_4_/Ti_3_C_2_ composite.

Figure 1 shows the synthesis process of the NiCo_2_O_4_/Ti_3_C_2_ composite. (1) The construction of Ti_3_C_2_/NF 3D structure via vacuum filtration. Firstly, 1 g Ti_3_AlC_2_ (Figure S1a, Supplementary Materials) was etched by using 30 mL HF (40 wt%) under magnetic stirring at 50 °C for 36 h. The resultant sediments were washed with deionized water and ethanol several times via centrifugation until pH reached neutrality and dried at 60 °C in an oven. The obtained accordion-like Ti_3_C_2_ powders (Figure S1b) were dispersed in 20 mL deionized water followed by sonicating for 20 min at ice-bath ambient and centrifuged at 3500 rpm for 1 h to gain the supernatant of few-layer Ti_3_C_2_ nanosheets. Then, Ni foams (NFs, 1 × 1.5 cm^2^) were cleaned in acetone, 6 M HCl, deionized water and ethanol for ultrasonic cleaning for 15 min, respectively, to remove the stains and oxides on their surface and dried for further use. Afterwards, few-layer Ti_3_C_2_ nanosheet supernatant was dropped on the pretreated NF with the assist of vacuum filtration to gain Ti_3_C_2_/NF 3D network skeleton structure. (2) The growth of NiCo_2_O_4_ on the surface of Ti_3_C_2_/NF was realized via a solvothermal process. 0.05 M Ni(NO_3_)_2_·6H_2_O, 0.1 M Co(NO_3_)_2_·6H_2_O and 0.42 M CH_4_N_2_O were dissolved in the solution consisted of 20 mL deionized water and 20 mL ethanol followed by magnetic stirring for 20 min to form a clear pink solution. Subsequently, the above obtained Ti_3_C_2_/NF was immersed into the solution, which was put in a Teflon-lined stainless-steel autoclave. The autoclave was heated at 150 °C for 4 h to grow Ni−Co precursor nanowires on Ti_3_C_2_/NF surface. Finally, the NiCo_2_O_4_/Ti_3_C_2_ composite was obtained after annealing at 350 °C for 2 h in N_2_ at a heating rate of 1 °C min^−1^ with the transform from precursor to NiCo_2_O_4_. The active material on NF in NiCo_2_O_4_/Ti_3_C_2_ is about 1.5 mg. Noteworthily, the abundant functional terminal groups (-F, -OH, -O, etc.) on the few-layer Ti_3_C_2_ surface will provide a large number of nucleation sites for in situ growth of NiCo_2_O_4_ nanowires as a result of electrostatic interaction between Co and Ni cations and negatively charged MXene [12]. For comparison, the NiCo_2_O_4_/NF and pure NiCo_2_O_4_ powders were synthesized by the same method. 

### 2.3. Instruments and Characterization

The structures and morphologies of electrodes were characterized by field emission scanning electron microscope (FE-SEM, FEI, Quanta 650 FEG, New York, America), and the detailed microstructure was further observed by transmission electron microscopy (TEM, JEOL, JEM-2100Plus, Osaka, Japan) at 200 KV. The elemental compositions were tested by energy dispersive X-ray spectroscopy (EDS). X-ray diffraction (XRD, Rigaku, Ultima IV, Osaka, Japan) was performed to investigate phase compositions using Cu Kα radiation (λ = 0.15418 nm). Surface valences were analyzed by X-ray photoelectron spectroscopy (XPS, Thermo Scientific, K-Alpha, New, York, NY, USA) with Al Kα radiation. Specific surface and pore information were measured by nitrogen adsorption–desorption isotherms (Micromeritics, ASAP 2460, Norcross, GA, USA) at 77 K. The specific surface area was calculated by the Brunauer–Emmett–Teller (BET) formula, and the pore size distribution was obtained by the Barret–Joyner–Halenda (BJH) method.

### 2.4. Fabrication of the NiCo_2_O_4_/Ti_3_C_2_//AC Asymmetrical Supercapacitor (ASC)

The AC/NF negative electrode was prepared by painting the uniform mixtures of activated carbon (AC), polyvinylidene fluoride (PVDF) binder and acetylene black conductive agent with the mass ratio of 8:1:1 on the pretreated NF. The ASC was assembled in a coin cell (CR2032) with the order of positive case, NiCo_2_O_4_/Ti_3_C_2_, electrolyte (6 M KOH), separator (Celgard 3501), electrolyte, AC/NF, spacer, spring, negative case, respectively.

### 2.5. Electrochemical Measurements

The electrochemical performance of the NiCo_2_O_4_/Ti_3_C_2_ electrode and the asymmetrical supercapacitor (ASC) was tested on an electrochemical workstation (CHI660E). For NiCo_2_O_4_/Ti_3_C_2_ electrode, the cyclic voltammetry (CV, 0–0.6 V), galvanostatic charge-discharge (GCD, 0–0.5 V) and electrochemical impedance spectroscopy (EIS, 5 mV, 100 KHz–0.01 Hz) curves were investigated by a three-electrode system consisting of platinum foil counter electrode, double salt bridge Hg/HgO reference electrode and NiCo_2_O_4_/Ti_3_C_2_ working electrode. For ACS, the electrochemical performance was measured by the electrode clamp connected to the electrochemical workstation. The CV (0–1.6 V) and GCD (0–1.6 V) curves were recorded at various scan rates of 10–100 mV s^−1^ and current densities of 1–10 A g^−1^, respectively. In addition, the specific capacitance of NiCo_2_O_4_/Ti_3_C_2_ in a three-electrode system and ACS was calculated from the GCD curves by Equations (1)–(3) [25,26,27]:

(1) $${\;C}_{m}=I\Delta t/m\Delta V$$

where *C_m_* (F g^−1^), *I* (A), Δ*t* (s), *m* (g), Δ*V* (V) are the gravimetric specific capacitance, load current, discharge time, the mass of active material on NF and working potential range of NiCo_2_O_4_/Ti_3_C_2_ electrode, respectively.

The volumetric specific capacitance was calculated using the Equation (2):

(2) $$C_{v}=I\Delta t/v\Delta V$$

where *C_v_* (F cm^−3^), *I* (A), Δ*t* (s), *v* (cm^−3^), Δ*V* (V) are the volumetric specific capacitance, load current, discharge time, the mass of active material on NF and working potential range of NiCo_2_O_4_/Ti_3_C_2_ electrode, respectively. The thickness of NiCo_2_O_4_/Ti_3_C_2_ is 1 mm. The volume of the NiCo_2_O_4_/Ti_3_C_2_ is 0.15 cm^−3^.

(3) $$C_{A}=I\Delta t/m\Delta V$$

where *C_A_* (F g^−1^), *I* (A), Δ*t* (s), *m* (g), Δ*V* (V) represent for the specific capacitance, load current, discharge time, total mass of active materials in NiCo_2_O_4_/Ti_3_C_2_ positive and AC/NF negative electrodes and working potential range of the ACS, respectively.

The specific capacitance of NiCo_2_O_4_/Ti_3_C_2_ and AC/NF based on CV curves at 10 mV s^−1^ was gained according to Equation (4) [26]. The mass ratio between NiCo_2_O_4_/Ti_3_C_2_ positive and AC/NF negative electrodes was determined using the Equation (5) [26] for optimizing the ACS:

(4) $${\;C}_{p}=A/ms\Delta V$$

*m*^+^/*m*^−^ = (*C_p_*^−^ × Δ*V*^−^)/(*C_p_*^+^ × Δ*V*^+^) (5)

where *C_p_* (F g^−1^), *A*, *m* (g), *s* (mV s^−1^), ∆*V* (V) represent for specific capacitance, the integrated area in CV curves, the mass of active material, scan rate and working potential range of the positive and negative electrodes, respectively. The mass of active material on NiCo_2_O_4_/Ti_3_C_2_ and AC/NF was 1.5 mg and 0.85 mg initially and was well adjusted to be about 1:1 (1.5 mg:1.5 mg) ultimately in this work.

The energy density (*E*, Wh kg^−1^) and power density (*P*, W kg^−1^) of ACS were obtained according to the Equations (6) and (7) [25,26]:

(6) $$E=0.5\;\times \;C_{A\;}\times \;(\Delta {V)}^{2}\;/\;3.6$$

(7) P=E × 3600 / Δt

where *C_A_* (F g^−1^), Δ*V* (V), Δ*t* (s) represent for specific capacitance, working potential range, discharge time of ACS.

## 3. Results and Discussion

### 3.1. Structural and Morphological Characterization

**Figure 2:**
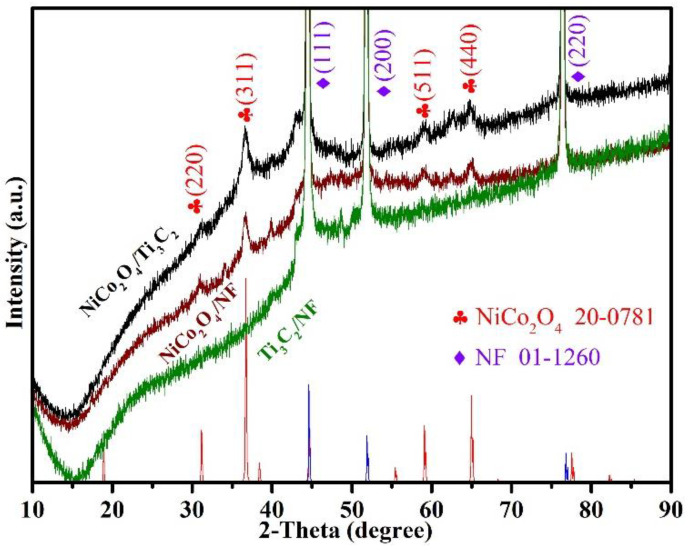
XRD patterns of Ti_3_C_2_/NF, NiCo_2_O_4_/NF, NiCo_2_O_4_/Ti_3_C_2_.

XRD patterns were performed to analyze the phase compositions of the samples. Figure S2 (Supplementary Materials) shows the XRD patterns of Ti_3_AlC_2_ and the etched accordion-like Ti_3_C_2_ powders. The disappearance of characteristic peak of (104) plane in Ti_3_AlC_2_ indicates the removal of Al layer after HF etching. Meanwhile, the (002) plane located at 8.6° in Ti_3_C_2_ is obviously broadened and shifts to a low angle compared with Ti_3_AlC_2_, implying a larger interlayer distance [28]. In Figure 2, besides the three strong peaks of NF, there are no diffraction peaks of Ti_3_C_2_ that can be observed in Ti_3_C_2_/NF, which is different from Ti_3_C_2_ powders due to the poor crystallinity of Ti_3_C_2_ [29]. The diffraction peaks located at 31.16°, 36.7°, 59.06° and 64.94° in NiCo_2_O_4_/NF are well assigned to the (220), (311), (511) and (440) planes of NiCo_2_O_4_ (JCPDS No. 20-0781) [30], respectively. As for NiCo_2_O_4_/Ti_3_C_2_, only the diffraction peaks of NiCo_2_O_4_ can be observed and no obvious peaks of Ti_3_C_2_ can be found due to the poor crystallinity of Ti_3_C_2_ or maybe the blocking effect of NiCo_2_O_4_ resulted from the full decoration of Ti_3_C_2_/NF by NiCo_2_O_4_.

**Figure 3:**
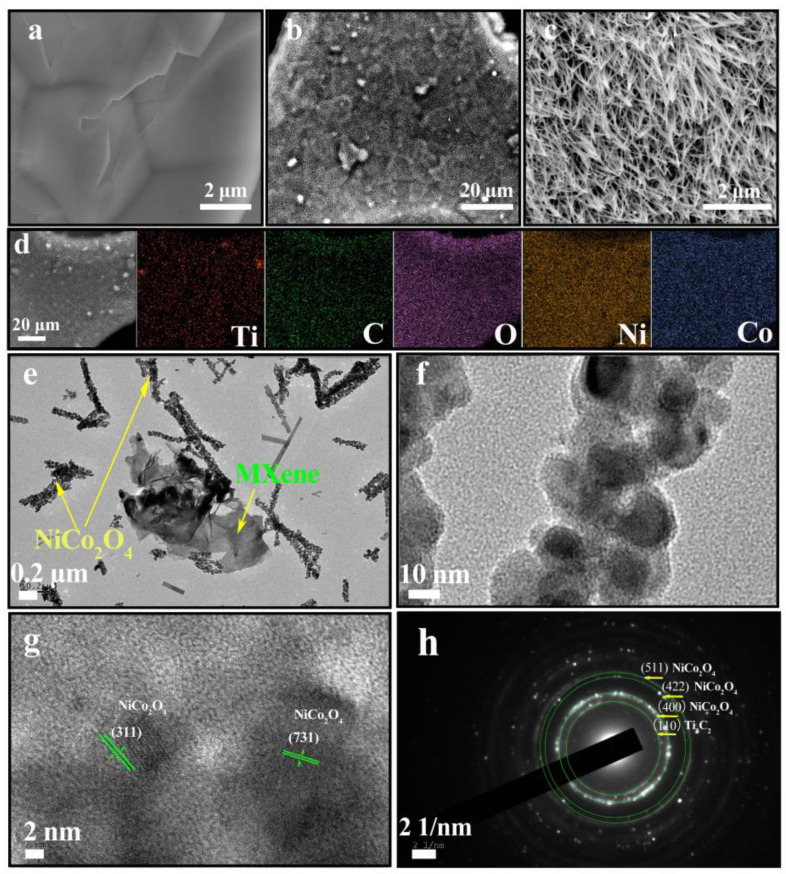
(**a**) SEM image of Ti_3_C_2_/NF, (**b**,**c**) SEM images and (**d**) elemental mapping of NiCo_2_O_4_/Ti_3_C_2_, (**e**–**g**) TEM images and (**h**) SADE pattern of NiCo_2_O_4_/Ti_3_C_2_.

The NiCo_2_O_4_/Ti_3_C_2_ and Ti_3_C_2_/NF samples were characterized by SEM in Figure 3. As observed in Figure 3a, graphene-like Ti_3_C_2_ nanosheets are successfully attached on NF surface, due to the strong physical adsorption between Ti_3_C_2_ and NF caused by vacuum filtration. The powerful vacuum filtration also contributes for the close connection between NF and Ti_3_C_2_, which is conducive to the electron transport and lower resistance in electrode [31]. From Figure 3b,c, it can be seen that the surface of Ti_3_C_2_/NF becomes rough when Ti_3_C_2_/NF is used as substrate to grow NiCo_2_O_4_, and Ti_3_C_2_ can hardly be found owing to the dense needle-like NiCo_2_O_4_ nanowires covering on its surface. However, it can still be deduced from the uneven surface that Ti_3_C_2_ nanosheets exist in the composite. The cross-section of NiCo_2_O_4_/Ti_3_C_2_ in Figure S3 further shows NiCo_2_O_4_ nanowires with a length of up to several micrometers vertically grow on Ti_3_C_2_/NF, while the pure NiCo_2_O_4_ powders possess an urchin-like microsphere structure as shown in Figure S4. The phenomenon indicates that the separately and uniformly dispersed NiCo_2_O_4_ nanowires on Ti_3_C_2_/NF will provide large specific surface area and abundant active sites for improving electrochemical performance. The element mapping (Figure 3d) clearly shows the existence and uniform distribution of Ti, C, O, Ni and Co elements in as-prepared composite electrode, which further reveals the successful synthesis of NiCo_2_O_4_/Ti_3_C_2_ nanostructure.

The active material of NiCo_2_O_4_/Ti_3_C_2_ was peeled from NF under ultrasound in a vacuum to further characterize the microstructure, as shown in Figure 3e–h. The peeling took a long time, indicating the strong contact of active material components, which is beneficial to the electron transport and structural stability of NiCo_2_O_4_/Ti_3_C_2_ and synergistic effects among components. As observed in Figure 3e, the broken NiCo_2_O_4_ nanowires are distributed around the few-layer Ti_3_C_2_ nanosheet. In Figure 3f, needle-like NiCo_2_O_4_ nanowires are composed of numerous nanoparticles, similar to the previous work [32], with a diameter of about 15 nm. Massive channels existing in nanoparticles will provide large surface area, which is beneficial to the sufficient contact between electrolyte and electrode material, and promote the rapid ions transport and pseudocapacitance reactions. As observed in Figure 3g, the interplanar spacings of 0.27 nm and 0.10 nm can be indexed to the (311) and (731) planes of NiCo_2_O_4_, respectively. In addition, SADE pattern in Figure 3h displays many diffraction rings with different diameters, which can be ascribed to the (400), (511), (531), (711) planes of NiCo_2_O_4_ showing the polycrystalline structure.

**Figure 4:**
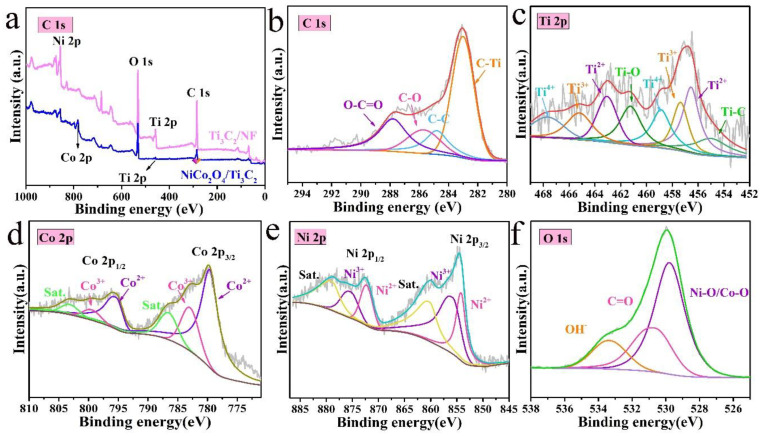
(**a**) XPS survey spectra of NiCo_2_O_4_/Ti_3_C_2_ and Ti_3_C_2_/NF, (**b**–**f**) typical fitted high-resolution spectra of C 1s, Ti 2p, Co 2p, Ni 2p and O 1s of NiCo_2_O_4_/Ti_3_C_2_.

The surface chemical states and elemental compositions of prepared materials were examined by XPS, as shown in Figure 4. In Figure 4a, the XPS survey spectra indicate Ni, Ti, C and O elements are presented in Ti_3_C_2_/NF, and a new peak with the binding energy of 782.1 eV corresponding to Co element appears in NiCo_2_O_4_/Ti_3_C_2_ except for the same elements as Ti_3_C_2_/NF, confirming that the NiCo_2_O_4_ has been loaded on the Ti_3_C_2_/NF surface. Moreover, Ti element peak in NiCo_2_O_4_/Ti_3_C_2_ is weakened, resulting from the complete coverage of NiCo_2_O_4_ on Ti_3_C_2_/NF, which is in agreement with the XRD. Fine XPS spectra of C 1s, Ti 2p, Co 2p, Ni 2p, and O 1s for NiCo_2_O_4_/Ti_3_C_2_ are presented in Figure 4b–f. The typical fitted C 1s spectrum in Figure 4b shows that the peaks located at 282.9 eV, 284.8 eV, 285.7 eV and 287.7 eV are assigned to C−Ti, C−C, C−O and O−C=O, respectively, which prove the presence of functional groups on Ti_3_C_2_ [33]. Ti 2p spectrum (Figure 4c) exhibits the peaks at 455.1 eV and 461.2 eV and these are attributed to Ti−C and Ti−O, respectively, and the peaks at 456.6/463.1 eV, 457.4/465.3 eV and 458.8/467.7 eV can be indexed to Ti^2+^, Ti^3+^ and Ti^4+^, showing the mixed valences of Ti_3_C_2_ [34,35]. In Co 2p spectrum (Figure 4d), the peaks at 779.6/795.8 eV and 783.2/799.8 eV can be indexed to Co^2+^ and Co^3+^ [36]. Similar to Co 2p, the Ni 2p spectrum (Figure 4e) possesses a typical doublet of Ni 2p_3/2_ (854.48 eV) and Ni 2p_1/2_ (872.58 eV) and broad shakeup satellites [36]. The multivalent valences of Ni and Co will make contributions to high specific capacitance in SCs. The spectrum of O 1s is shown in Figure 4f. The peaks at 529.7 eV, 530.7 eV and 533.4 eV correspond well to the Co−O or Ni−O, C=O and OH^−^, respectively [21,33].

**Figure 5:**
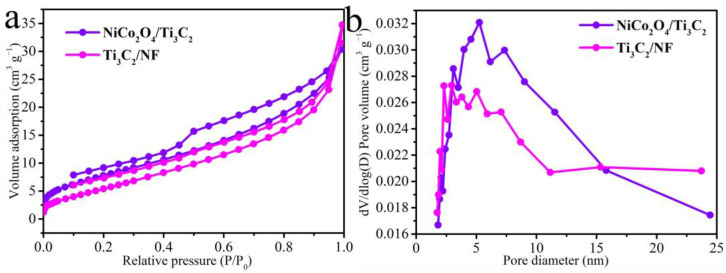
(**a**) N_2_ adsorption–desorption isotherms, (**b**) pore diameter distributions of NiCo_2_O_4_/Ti_3_C_2_ and Ti_3_C_2_/NF.

N_2_ adsorption–desorption isotherms were conducted to demonstrate the specific surface area and pore diameter distributions of the NiCo_2_O_4_/Ti_3_C_2_ and Ti_3_C_2_/NF. NiCo_2_O_4_/Ti_3_C_2_ displays a distinct type IV hysteresis loop in Figure 5a, indicating the presence of mesoporous structure and a higher calculated specific surface area (35.2 m^2^ g^−1^) compared to Ti_3_C_2_/NF (20.7 m^2^ g^−1^). Moreover, the pores size values in Figure 5b analyzed by the BJH method further show that the pore volume of NiCo_2_O_4_/Ti_3_C_2_ is higher than Ti_3_C_2_/NF, which can be mainly attributed to the massive mesoporous structure of NiCo_2_O_4_. Porous structure and large active surface in NiCo_2_O_4_/Ti_3_C_2_ both promote ions or electron transport and penetration of electrolyte, optimizing the electrochemical performance [37].

### 3.2. Electrochemical Performances of Samples

**Figure 6:**
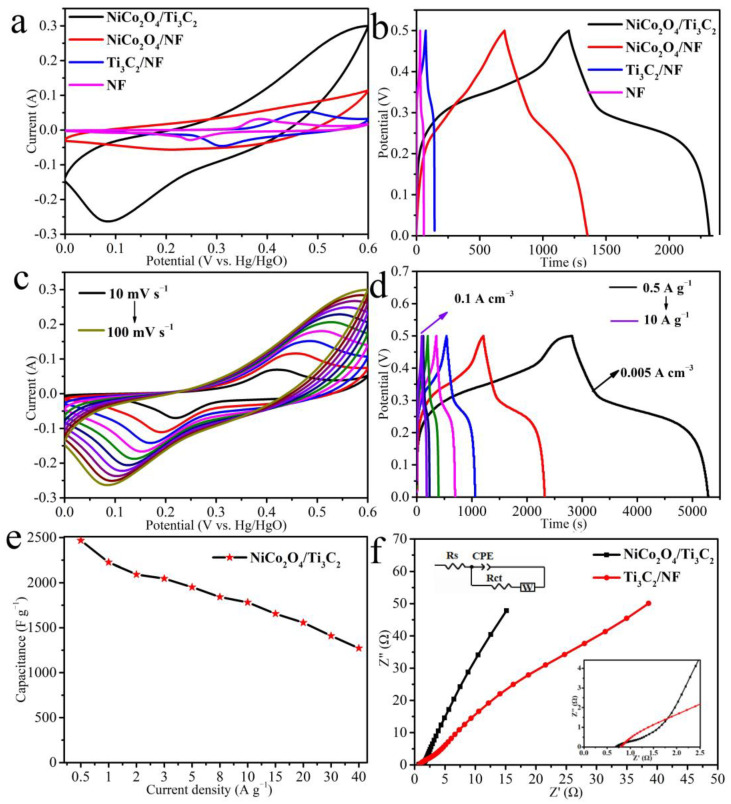
(**a**) CV curves at 100 mV s^−1^ and (**b**) GCD curves at 1 A g^−1^ of the obtained samples, (**c**) CV curves and (**d**) GCD curves of NiCo_2_O_4_/Ti_3_C_2_, (**e**) specific capacitance and (**f**) Nyquist plots of NiCo_2_O_4_/Ti_3_C_2_ and Ti_3_C_2_/NF.

The electrochemical performance of the as-prepared samples was investigated using a three-electrode system in 6 M KOH as illustrated in Figure 6. Firstly, the performance comparison of NF, Ti_3_C_2_/NF, NiCo_2_O_4_/NF and NiCo_2_O_4_/Ti_3_C_2_ is carried out to exclude the effect of NF as substrate on capacitance, as shown in Figure 6a,b. It is well known that the larger integrated area of CV curves and longer charge–discharge time of GCD reflect higher specific capacitance. Thus, it can be deduced from Figure 6a,b that the contribution of NF to capacitance of electrodes is very little. Furthermore, it can be seen that the introductions of Ti_3_C_2_ and NiCo_2_O_4_ into NF result in higher current signal and specific capacitance mainly due to their mutually synergistic effects, compared with Ti_3_C_2_/NF and NiCo_2_O_4_/NF. 

The electrochemical performance of NiCo_2_O_4_/Ti_3_C_2_ is studied in detail, as observed in Figure 6c–f. Figure 6c shows the CV curves at different scan rates from 10 mV s^−^^1^ to 100 mV s^−^^1^. Each curve possesses a pair of redox peaks, which can be attributed to the faradaic reactions of M−O/M−O−OH (M = Co, Ni) [38]. With the increase of scan rate, the approximately same CV shape indicates the good rate performance of NiCo_2_O_4_/Ti_3_C_2_. The oxidation peak and reduction peak shift to the positive range and negative range of values, respectively, which may result from the polarization effect of electrode material [39]. The GCD curves at various current densities of NiCo_2_O_4_/Ti_3_C_2_ ranging from 0.5 A g^−^^1^ to 10 A g^−^^1^ are shown in Figure 6d. It can be seen that the discharge time at 0.5 A g^−^^1^ can even up to 2500 s, which is almost longest among previous reports. The approximately triangular and symmetrical charge–discharge curves can still be kept even at 10 A g^−^^1^, indicating the rapid I−V response and high charge-discharge reversibility of NiCo_2_O_4_/Ti_3_C_2_ [40]. Moreover, the corresponding gravimetric specific capacitances (*C_m_*) and volumetric specific capacitances (*C_p_*) are calculated by Equations (1) and (2) in Figure 6e. The gravimetric specific capacitance of 2468 F g^−1^ and volumetric specific capacitances of 24.7 F cm^−3^ at 0.5 A g^−1^ are gained, and 1782 F g^−1^ (*C_m_*) and 17.8 F cm^−3^ (*C_p_*) are obtained at 10 A g^−1^, both with a high capacitance retention of 72%, demonstrating the good rate performance of composite.

Nyquist plots of EIS spectrum will provide the information about the electrochemical reaction kinetics of electrode. A semicircle in high frequency region and a straight line in low frequency region are comprised in a Nyquist plot. The diameter of semicircle is related to the charge transfer resistance value (R_ct_), the straight line is interpreted as the Warburg resistance (Z_w_), reflecting the ions diffusion resistance in the electrode, and the intercept with the real axis represents the equivalent series resistance (R_s_) [41,42]. As shown in Figure 6f, the R_s_ of NiCo_2_O_4_/Ti_3_C_2_ (0.69 Ω) is lower than that of Ti_3_C_2_/NF (0.81 Ω) on the basis of equivalent circuit [43], which mainly results from quick electrolyte penetration into massive channels of NiCo_2_O_4_. Simultaneously, these channels and the top sharp ends of nanowires can easily capture ions, decreasing the ions’ diffusion resistance, as explained by a more vertical line.

**Figure 7:**
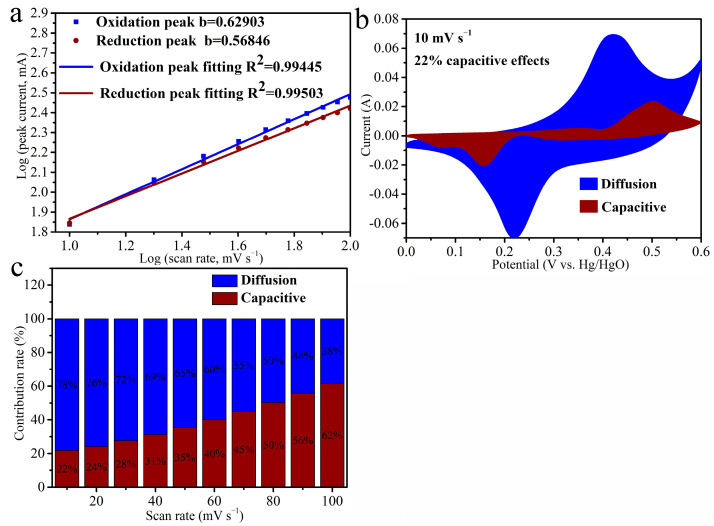
(**a**) The relationship between peak currents and scan rates in charge and discharge process, (**b**) contribution of surface capacitive process at 10 mV s^−1^, (**c**) contribution proportions of surface capacitive process at various scan rates of NiCo_2_O_4_/Ti_3_C_2_.

The storage mechanism of NiCo_2_O_4_/Ti_3_C_2_ electrode is explored based on the CV curves. The contributions of surface capacitive process and diffusion-controlled process are analyzed according to the b value in formula i = av^b^ [44,45], where i represents peak current, v signifies scan rate. “b = 1” declares a surface capacitive process and “b = 0.5” describes a diffusion-controlled process. As shown in Figure 7a,b, values in charge and discharge process of NiCo_2_O_4_/Ti_3_C_2_ are 0.629 and 0.568, respectively, indicating that the energy storage is determined by both capacitive process and diffusion-controlled process. The quantitative contributions of surface capacitive process and diffusion-controlled process at different scan rates are analyzed based on formula i(V) = k_1_v + k_2_v^1/2^ [46], where k_1_v and k_2_v^1/2^ represent the currents from surface capacitive process and diffusion-controlled process, respectively. Figure 7b shows that the contribution of surface capacitive process at 10 mV s^−1^ is approximately 22%. Moreover, the contribution proportions of surface capacitive process (Figure 7c) show a constant rising tendency with the increase of scan rate because of the diffusion delay of electrolyte ions to internal electrode, leading to the decrease of the contribution of the diffusion-controlled process [47].

NiCo_2_O_4_ nanowires composed with massive nanoparticles vertically grow on 3D Ti_3_C_2_/NF skeleton in NiCo_2_O_4_/Ti_3_C_2_. The excellent electrochemical performance of NiCo_2_O_4_/Ti_3_C_2_ can be attributed to its unique structure feature. The excellent capacitance performance of NiCo_2_O_4_/Ti_3_C_2_ in a three-electrode system is compared with other carbon-based electrodes and metal oxides/sulfides-based electrodes in previous works [48,49,50,51,52,53,54,55] and electrodes with similar morphology (Gd/CeOx nanoflowers/porous carbon [56], Co_3_O_4_ nanosheets/carbon foam [57]). The comparison results are listed in Table S1 [48,49,50,51,52,53,54,55,56,57]. It is worth emphasizing that the results in our work are in a high level overall, especially the specific capacitance (2468 F g^−1^ at 0.5 A g^−1^, 2226 F g^−1^ at 1 A g^−1^) is even superior to other works. Firstly, the direct contact without binders among components and amounts of micropores of NiCo_2_O_4_ effectively reduce the interface resistance, and promote ions and electron transport. Moreover, Ti_3_C_2_ nanosheets can make up for the conductivity of NiCo_2_O_4_ by a close contact leading to a continuous electron pathway. Electron transport in the internal electrode is accelerated. Thirdly, NiCo_2_O_4_ nanowires vertically grow on the Ti_3_C_2_/NF substrate rather than the agglomerated urchin behavior, which will reduce the “dead volume” and further improve electrochemical performance. What is more, the situ-growth of NiCo_2_O_4_ on Ti_3_C_2_/NF incites a robust heterostructure, improving the cycle performance of NiCo_2_O_4_/NF.

### 3.3. Electrochemical Performances of the ASC

With the destination of investigating the electrochemical performance of NiCo_2_O_4_/Ti_3_C_2_ electrode applied in supercapacitors, an NiCo_2_O_4_/Ti_3_C_2_//AC ASC was established. Initially, the CV curves of AC/NF negative electrode (Figure S5a) were conducted in a three-electrode system to detect the appropriate potential window. It can be seen that AC/NF shows polarization in CV curves of −1.1–0 V and −1.2–0 V. Moreover, an approximate rectangle without polarization can be observed at −1–0 V, indicating the double-electric-layer energy storage of AC, which also can be proved by the triangle shapes in GCD curves (Figure S5b). The high rate performance and brilliant kinetics behavior of AC/NF were evaluated by CV curves at various scan rates (10–100 mV s^−^^1^) and GCD curves at various current densities (0.5–40 A g^−1^) at −1–0 V (Figure S5c,d) to ensure its superiority and eliminate its negative effects to ASC.

**Figure 8:**
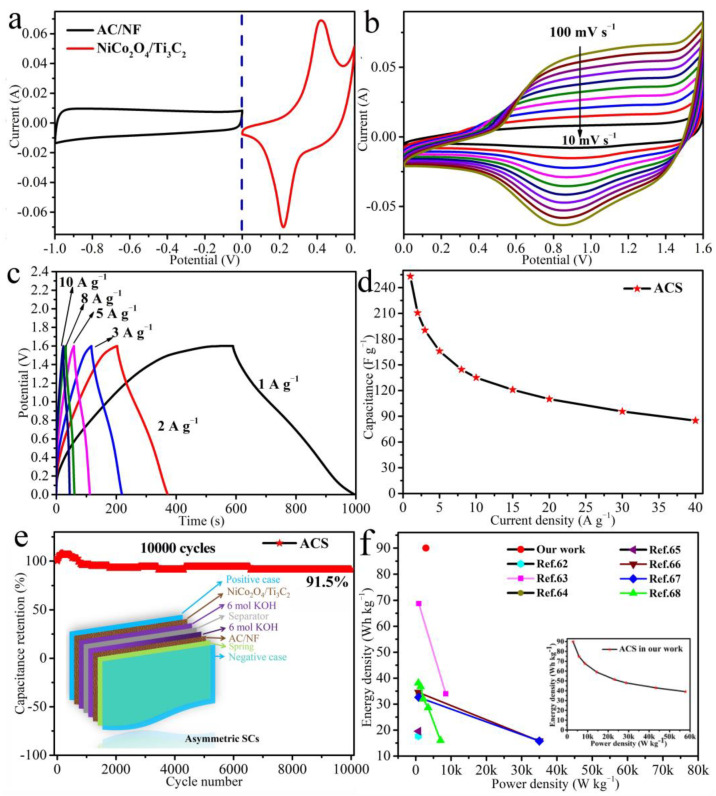
(**a**) CV curves of NiCo_2_O_4_/Ti_3_C_2_ and AC/NF in a three-electrode system, (**b**) CV curves, (**c**) GCD curves and (**d**) specific capacitance at various current densities of ACS, (**e**) cyclic stability of ACS at 15 A g^−1^ for 10,000 cycles, (**f**) the Ragone plots of the Ti_3_C_2_ and Ni−Co based supercapacitors in previous works (inset is the Ragone plot of ACS in our work).

The CV curves of NiCo_2_O_4_/Ti_3_C_2_ (0–0.6 V vs. Hg/HgO) and AC/NF (−1–0 V vs. Ag/AgCl) at 10 mV s^−1^ in a three-electrode system are displayed in Figure 8a. The pseudocapacitance characteristics of NiCo_2_O_4_/Ti_3_C_2_ with redox peaks and the electrical double-layer characteristics of AC/NF with quasi-rectangle are coupled with each other and displays an irregular shape, as shown in Figure 8b, suggesting the mutual contribution of EDLC- and battery-type electrodes to the total capacitance [41]. To our knowledge, extensive potential window will not only improve the energy density but also increase the output potential of supercapacitors, which will push them towards a more practical use. For further determining and optimizing the potential window of ACS, the CV curves (0–1 V to 0–1.9 V) and the GCD curves (0–1 V, 0–1.2 V, 0–1.4 V, 0–1.6 V with 0.2 V steps increasing) were tested (Figure S6a,b). It can be observed that the phenomenon of polarization becomes obvious with the increase in potential window from Figure S6a, and the optimal potential window is considered to be 0–1.6 V. Moreover, the GCD curves further confirm the accessibility of 0–1.6 V (Figure S6b), which is ultimately certified as the working potential window of ACS for further study. 

Figure 8b presents the CV curves of the ACS device at various scan rates from 10 mV s^−1^ to 100 mV s^−1^. The similar shape and the increased area of the CV curves can be found with the scan rate increasing, suggesting the fast transport of ions and electron at the electrode/electrolyte interfaces and the good high-current charge/discharge performance within the device [58]. The GCD curves ranging from 1 A g^−1^ to 10 A g^−1^ at a high potential of 0–1.6 V show approximately triangular-like and symmetrical shape and the negligible IR drop (Figure 8c), manifesting the ideal capacitive behavior [59], the high coulombic efficiency [60] and the low IR of the device [61]. The corresponding specific capacitances as a function of current densities are calculated by Equation (3), as shown in Figure 8d. The specific capacitance can reach up to 253 F g^−1^ at 1 A g^−1^, and it decreases with current density increasing due to the delay of ions diffusion. The specific capacitance is 135 F g^−1^ with a retention of 53% at a high current density of 10 A g^−1^. 

The cyclic life is a critical factor for the device and was evaluated at 15 A g^−1^ for 10,000 cycles, as depicted in Figure 8e. The capacitance increases slightly at first due to the activation progress of the electrode [3], and tends to be steady over 700 cycles, and ultimately retains as high as 91.5% of its initial value, revealing an excellent cyclic stability. The excellent cyclic performance is on account of the close contact among active materials in NiCo_2_O_4_/Ti_3_C_2_ and the robust Ti_3_C_2_/NF substrate, holding an interlaced network structure with outstanding structural stability, which can support the NiCo_2_O_4_ nanowires and avoid their collapse and agglomeration in the cycling process. SEM images of NiCo_2_O_4_/Ti_3_C_2_ before and after cycles are displayed in Figure S7. By comparison, no significant change is found over 10,000 cycles, and the nanowires structure can be maintained well but only some white knots generating on the top of NiCo_2_O_4_ nanowires, leading to the decrease in the active surface and little loss of capacitance (about 8.5%). Energy density and power density are further calculated and plotted to evaluate the device performance for practical applications. From the inset of Figure 8f, the ultra-high energy densities up to 90, 74.84, 67.67, 59, 51.38, 48 Wh kg^−1^ can be achieved, when the power densities are 2880, 5760, 8640, 14,400, 23,119.2, 28,800 W kg^−1^ at 1, 2, 3, 5, 8, 10 A g^−1^, respectively. The presented energy density is compared with other reported ASCs based on Ti_3_C_2_ and Ni−Co materials in previous works (Figure 8f), such as Ti_3_C_2_T_x_/NiS//C/AC (17.688 Wh kg^−1^ at 750 W kg^−1^) [62], NiCo_2_S_4_/MXene//AC (68.7 Wh kg^−1^ at 850 W kg^−1^) [63], MXene/NiCoZDH//AC (34 Wh kg^−1^ at 748.6 W kg^−1^) [64], NiCo_2_O_4_@Au//AC/NF (19.56 Wh kg^−1^ at 782.59 W kg^−1^) [65], NiCo_2_O_4_/rGO/CNT//AC (34.5 Wh kg^−1^ at 799 W kg^−1^) [66], NiCo_2_O_4_/Ni wire//Fe_3_O_4_/Ni wire (32.6 Wh kg^−1^ at 846 W kg^−1^) [67], NiCo_2_S_4_//AC (38.1 Wh kg^−1^ at 700 W kg^−1^) [68]. By comparison, the ACS in our work exhibits relatively high and comparable energy density and power density simultaneously, which manifests the NiCo_2_O_4_/Ti_3_C_2_ great potential in the construction of ASCs.

## 4. Conclusions

The NiCo_2_O_4_/Ti_3_C_2_ composite was constructed by the two-step method of vacuum filtration and solvothermal treatment using NF as substrate. The close and direct contact among vertical hierarchical layers is in favor of the electron transport, and the porous structure of the obtained NiCo_2_O_4_/Ti_3_C_2_ provides abundant conductive channels and active areas, which can reduce ions diffusion resistance and improve kinetic condition. Due to the synergistic effects of hierarchical components, the NiCo_2_O_4_/Ti_3_C_2_ possesses a high specific capability (2468 F g^−1^ at 0.5 A g^−1^ and 1782 F g^−1^ at 10 A g^−1^) and a good rate capability. Based on this, a high-performance ACS device based on NiCo_2_O_4_/Ti_3_C_2_ positive and AC/NF negative electrodes was assembled. The ACS device exhibits a competitive capacitance of 253 F g^−1^ at 1 A g^−1^ and 135 F g^−1^ at 10 A g^−1^ with a relatively high retention of 53%. Moreover, only 8.5% capacitance loss is discovered after 10,000 cycles at 15 A g^−1^. More importantly, the brilliant energy density of 90, 74.84, 67.67, 59, 51.38, 48 Wh kg^−1^ are achieved at the power density of 2880, 5760, 8640, 14,400, 23,119.2, 28,800 W kg^−1^, respectively. This work provides an approach for the design and development of binder-free electrode in high performance energy storage application.

## Data Availability

The data can be provided upon request from the authors.

## Supplementary Materials

The following supporting information can be downloaded at: https://www.mdpi.com/article/10.3390/molecules27196452/s1, Figure S1: SEM images of (a) Ti_3_AlC_2_, (b) accordion-like Ti_3_C_2_; Figure S2: XRD patterns of Ti_3_AlC_2_ and Ti_3_C_2_; Figure S3: (a,b) SEM images of NiCo_2_O_4_/Ti_3_C_2_; Figure S4: SEM image of pure NiCo_2_O_4_; Figure S5: (a,b) CV and GCD curves at various potential windows, (c) CV curves at various scan rates, (d) GCD curves at various current densities of AC/NF; Figure S6: (a,b) CV and GCD curves of ACS at various potential windows; Figure S7: (a,b) Morphology before and after 10,000 cycles of NiCo_2_O_4_/Ti_3_C_2_; Table S1: Electrochemical performances of carbon-based electrodes and metal oxides/sulfides based electrodes and electrodes with similar morphology for supercapacitor application in a three-electrode system.
