# Supplementary material for: Facile Synthesis of NiCo2O4 Nanowire Arrays/Few-Layered Ti3C2-MXene Composite as Binder-Free Electrode for High-Performance Supercapacitors
Source: Molecules. 2022 Sep 30;27(19):6452. doi: 10.3390/molecules27196452 (PMC9572776; doi:10.3390/molecules27196452)
Supplement: Supplementary file 1 [file molecules-27-06452-s001.zip › molecules-1923218-supplementary.pdf]

Supplementary Material

Facile Synthesis of $NiCo_2O_4$ Nanowire Arrays/Few-Layered Ti_3C_2-MXene Composite as Binder-Free Electrode for High-Performance Supercapacitors

Yanhua Li [1,2], Shuhuan Wang [1,2], Guolong Ni [1,2], Qun Li [1,2,*]

[1] School of Metallurgy and Energy, North China University of Science and Technology, Tangshan 063210, China

[2] Key Laboratory of Special Metallurgy and Material Manufacture, Tangshan 063210, China

* Corresponding author. E-mail addresses: liq@ncst.edu.cn.

Table of Contents

1. SEM of Ti_3AlC_2, accordion-like Ti_3C_2
2. XRD patterns of Ti_3AlC_2, Ti_3C_2
3. SEM of $NiCo_2O_4/Ti_3C_2$
4. SEM of pure $NiCo_2O_4$ powders
5. Electrochemical performances of carbon-based electrodes and metal oxides/sulfides-based electrodes and electrodes with similar morphology for supercapacitor application in a three-electrode system.
6. CV and GCD curves of AC/NF
7. CV and GCD curves of ACS at various potential windows
8. Morphology before and after 10000 cycles of $NiCo_2O_4/Ti_3C_2$.

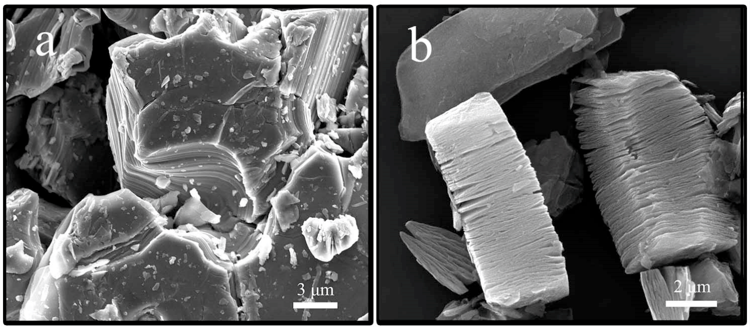

Figure S1. SEM images of (a) Ti_3AlC_2, (b) accordion-like Ti_3C_2.

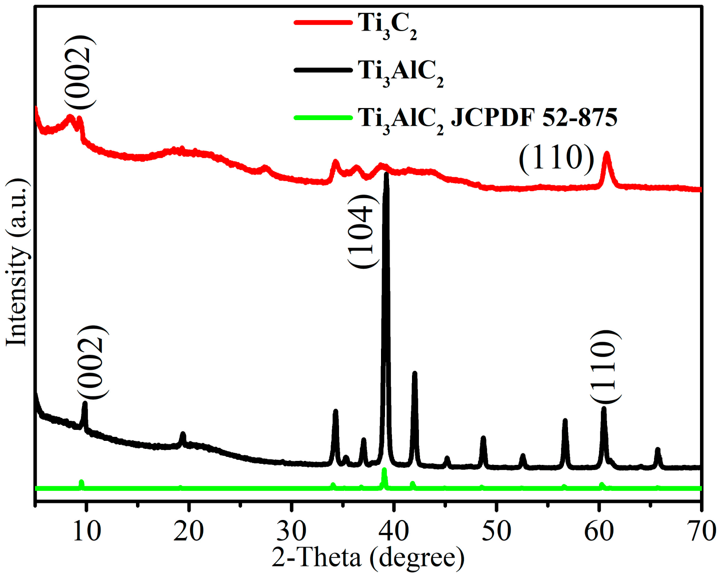

Figure S2. XRD patterns of Ti_3AlC_2 and Ti_3C_2.

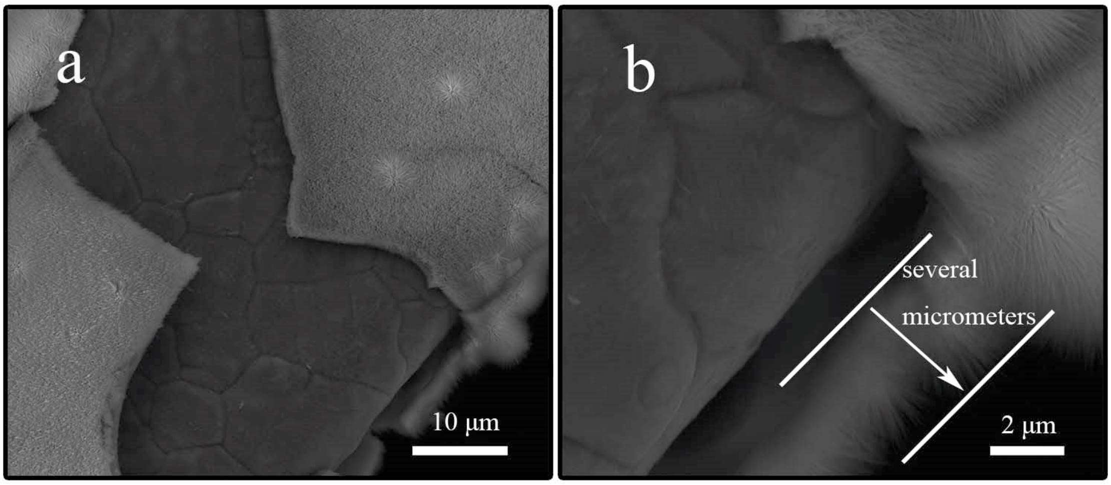

Figure S3. (a-b) SEM images of $NiCo_2O_4/Ti_3C_2$.

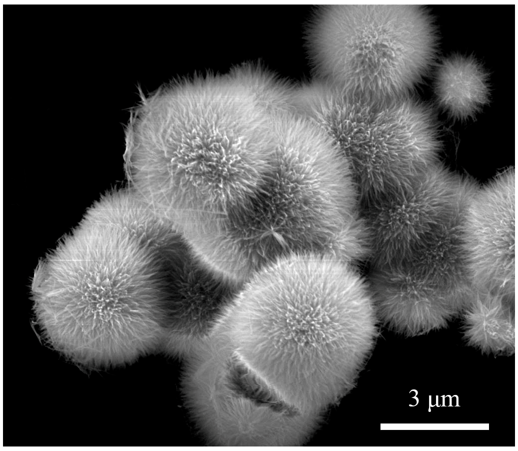

Figure S4. SEM image of pure $NiCo_2O_4$.

Table S1. Electrochemical performances of carbon based electrodes and metal oxides/sulfides based electrodes and electrodes with similar morphology for supercapacitor application in a three-electrode system.

Electrode material	Electrolyte	Potential (V)	Capacitance	Rate capability	Ref
**$Ti_3C_2T_x/NiCo_2S_4$@CC**	6 M KOH	0–0.7	2326 F g^{-1} at 1 A g^{-1}	66.3% (15 A g^{-1})	[48]
**P-Ti_3C_2@$NiCo_2S_4$**	3 M KOH	−0.1–0.4	973 F g^{-1} at 1 A g^{-1}	_	[49]
**Ti_3C_2/Ni-Co-Al-LDH**	1 M KOH	−0.2–0.5	1990 F g^{-1} at 1 A g^{-1}	_	[50]
**$NiCo_2O_4$@rGO/ACF**	3 M KOH	−0.2–0.6	1487 F g^{-1} at 3 mA cm^{-2}	60.45% (70 mA cm^{-2})	[51]
**NHCS/Ni_2CoS_4**	2 M KOH	0–0.5	1465 F g^{-1} at 0.5 A g^{-1}	57.33% (8 A g^{-1})	[52]
**N/S-doped porous carbons**	6 M KOH	−1–0	318 F g^{-1} at 0.5 A g^{-1}	71.7% (30 A g^{-1})	[53]
**V/$Co_{0.85}$Se**	3 M KOH	0–0.6	1.28 mA h cm^{-2} at 5 mA cm^{-2}	60.2% (30 mA cm^{-2})	[54]
**MnO_2@$SrCo_{0.875}Nb_{0.125}O_3$@CC**	0.5 M $MnCl_2$ and 0.5 M KNO_3	−0.4–0.6	2066.0 mF cm^{-2} at 2 mA cm^{-2}	59.2% (6 mA cm^{-2})	[55]
**Gd-doped CeOx/C**	1M KOH	0–1	280 F g^{-1} at 0.33A g^{-1}	_	[56]
**Co_3O_4/CC**	1M NaOH	0–1	106 F g^{-1} at 500 mV s^{-1}	_	[57]
**$NiCo_2O_4/Ti_3C_2$**	**6 M KOH**	**0–0.6**	**2468 F g^{-1} at 0.5 A g^{-1}**	**72% (10 A g^{-1})**	**This work**

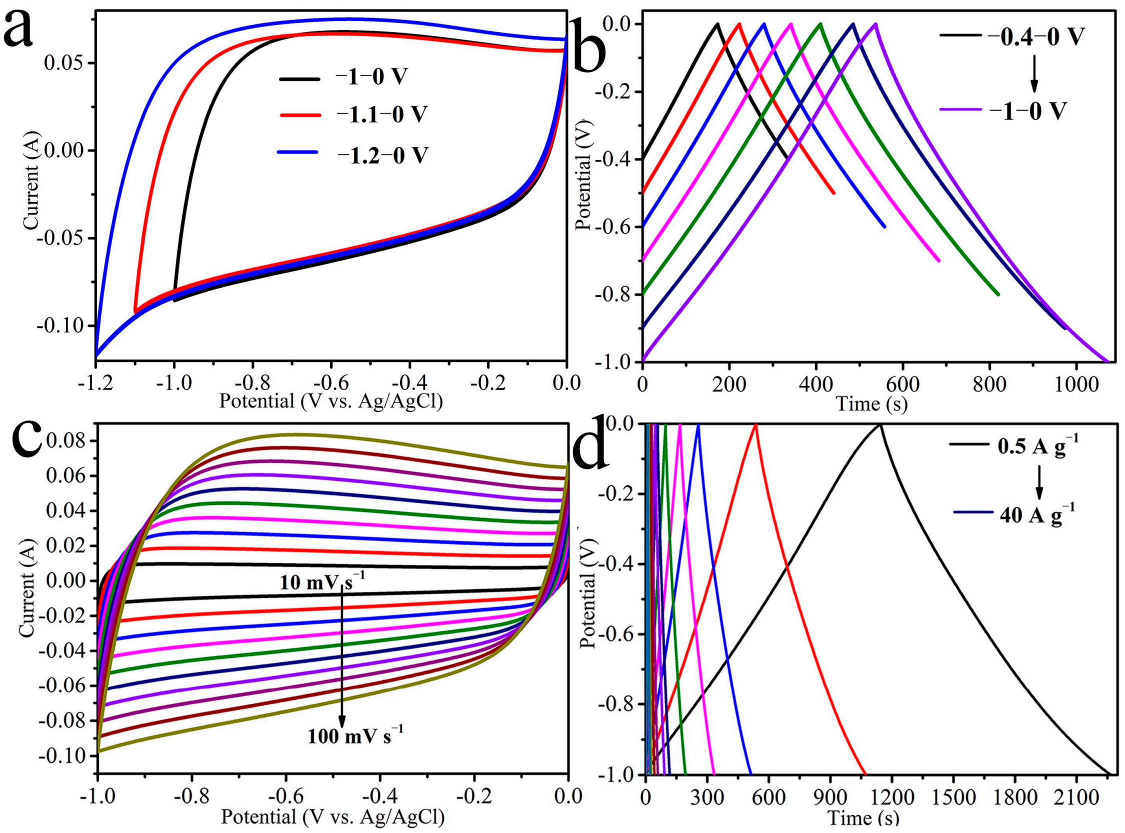

Figure S5. (a-b) CV and GCD curves at various potential windows, (c) CV curves at various scan rates, (d) GCD curves at various current densities of AC/NF.

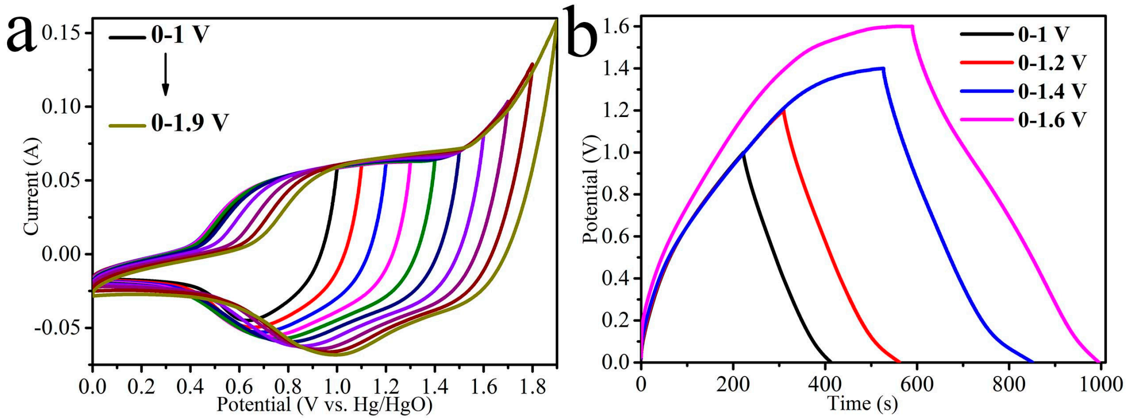

Figure S6. (a-b) CV and GCD curves of ACS at various potential windows.

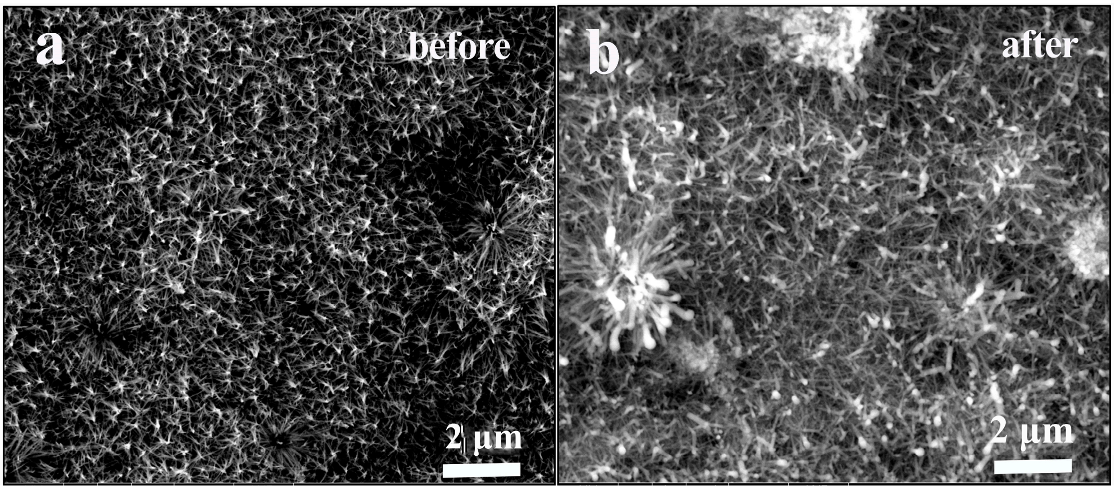

Figure S7. (a-b) morphology before and after 10,000 cycles of $NiCo_2O_4/Ti_3C_2$.
